# Patient safety in low-income countries

**Published:** 2015

**Authors:** Robert Lindfield

**Affiliations:** Clinical Lecturer: London School of Hygiene and Tropical Medicine; Consultant in Public Health: Public Health England, UK. Robert.Lindfield@lshtm.ac.uk

**Figure:**
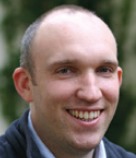
Robert Lindfield

The concept of ‘first do no harm’ is taught to every medical and nursing student. This phrase means that, as professionals, our first duty is to make sure that patients are not harmed as a result of their care.

Unfortunately, we know that many patients are harmed when receiving medical care. The World Health Organization (WHO) estimates that up to 10% of patients in high-income countries are harmed in such ‘adverse events’ or ‘critical incidents’ – events or incidents that caused harm to patients and could have been avoided.^1^

There are many costs associated with harm – costs to the patient in pain, discomfort or distress, financial costs to the patient (e.g. through increased visits to the hospital) and financial costs to the health care system, with patients staying in hospital longer and/or requiring more – and different – treatments. There is also the emergence of legal costs as increasing numbers of patients are suing the hospital or clinician following a critical incident.

The large numbers of patients harmed and the resultant costs make a focus on patient safety incredibly important. There are two ways of approaching patient safety:

1. Preventing critical incidents.
2. Learning from critical incidents that have occurred and changing practice so that it does not happen again.

**Figure:**
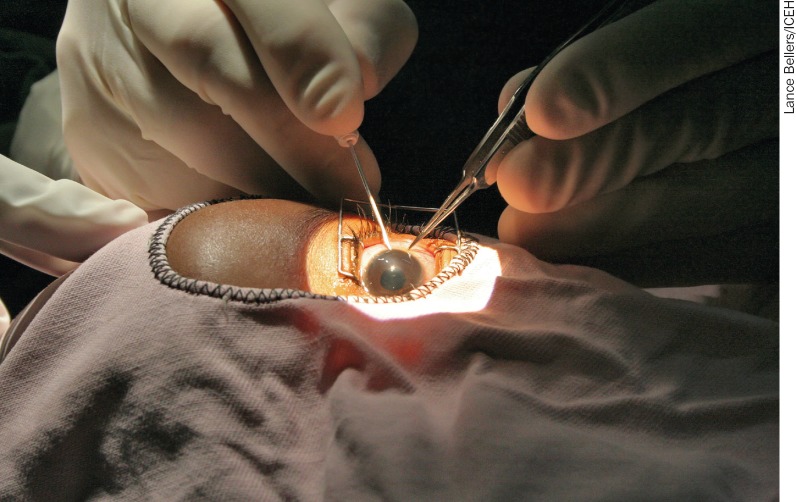
The Safety of our patients should be our first priority. ETHIOPIA

## 1 Preventing critical incidents

This is a focus on preventing harm. It involves the introduction of processes and procedures that reduce the risk of harm to patients. For example; everyone who conducts surgery changes clothes and washes their hands before they start which isa way of preventing harm. By washing hands (‘scrubbing’) and changing clothes before surgery the risk of passing infection to the patient is reduced. Other examples of prevention activities include the WHO safe surgical checklist (which aims to ensure that the correct surgery is being performed on the correct patient using the correct methods and provides a formal method to check this), staff double-checking doses of drugs, making sure that all equipment is regularly maintained, sterilising instruments, plus many others.

**ABOUT THIS ISSUE**

This issue of the *Community Eye Health Journal* is about patient safety. Ironically, when the focus of our day-to-day work is on improving the eye health of patients, it can be easy to overlook the central importance of safety. It is everyone in the eye team's responsibility to ensure that the day-to-day care we provide is safe and also patient-centred, so that patients will not just **be** safe, but also **feel** safe. Mistakes will happen – we are human, and humans make mistakes – but our task is to create systems and practices that reduce the risk of such ‘critical incidents’ taking place, and help us to learn from them if they do. This can only happen if everyone feels safe enough to report problems as they arise. We hope that this issue will help you to take time out from your daily routine to reflect on safety in all its aspects.

Two of the key factors about these procedures are that they are routine (i.e. everybody does them every time the event occurs) and that they can be documented (e.g. the WHO Safe Surgical Checklist is completed and kept with the patient notes) so that it can be shown that the procedure was followed. If the procedures and protocols are only followed by some of the people, some of the time, then they are less effective. A good example of this is hand hygiene practice amongst staff: this has been shown to be variable in many hospitals, exposing patients and staff to the transmission of infection.

## 2 Learning from critical incidents

Even in the best clinics and hospitals, patients are sometimes harmed. It is important that medical personnel learn from any critical incidents and put things in place to stop the same incident from occurring again (and therefore preventing further patients from being harmed).

One of the key principles when learning from harm is ‘no blame’. Our usual reaction when something goes wrong is to try and find someone to blame. However in many cases there are wider issues that have led to the mistake or incident.

Understanding the reasons behind mistakes helps the hospital to put procedures and protocols in place to try and prevent them from happening again. In the example (see panel) it might have been necessary to ensure that a specific number of staff members were on the ward on days when surgery was taking place; or that the IOL power was double-checked by the surgeon and theatre staff before being inserted into the eye.

Effective leadership is needed so that the staff members can learn from mistakes and make the hospital safer. There must be a system for investigating adverse events and finding out why they happened (page 26). Investigation should be done with the co-operation of the whole eye team because the solutions that make critical incidents less likely in the future usually require people to behave differently. If staff members see the reason for the change then they are more likely to adopt it. Blaming them, instead of exploring the issues that led to the incident, is likely to lead to a culture of fear where people try to hide problems and do not report critical incidents.

**Example**

The wrong intraocular lens is inserted in a patient's eye, leaving him with very poor vision despite successful surgery.

Whose fault is it? The surgeon? The nurse? The person who recorded the biometry?

Finding out the reason for the mistake might identify staff members who may have been at fault but it is equally important to understand why they made an error. Were there too many patients and too few staff members? Was the patient elderly, making it difficult to conduct biometry? Were the theatre staff rushing because the surgeon arrived late for a busy operating list?

The articles in this issue explore different aspects of patient safety and provide ideas about how harm to patients can be prevented, including harm from endophthalmitis. There is also a focus on keeping yourself safe and healthy-without this, safe patient care is not possible.
